# A Self-Adaptive Gallery Construction Method for Open-World Person Re-Identification

**DOI:** 10.3390/s23052662

**Published:** 2023-02-28

**Authors:** Sara Casao, Pablo Azagra, Ana C. Murillo, Eduardo Montijano

**Affiliations:** Department of Computer Science and Systems Engineering, Universidad de Zaragoza, 50018 Zaragoza, Spain

**Keywords:** person recognition, open-world recognition, incremental clustering

## Abstract

Person re-identification, or simply re-id, is the task of identifying again a person who has been seen in the past by a perception system. Multiple robotic applications, such as tracking or navigate-and-seek, use re-identification systems to perform their tasks. To solve the re-id problem, a common practice consists in using a *gallery* with relevant information about the people already observed. The construction of this gallery is a costly process, typically performed offline and only once because of the problems associated with labeling and storing new data as they arrive in the system. The resulting galleries from this process are static and do not acquire new knowledge from the scene, which is a limitation of the current re-id systems to work for open-world applications. Different from previous work, we overcome this limitation by presenting an unsupervised approach to automatically identify new people and incrementally build a gallery for open-world re-id that adapts prior knowledge with new information on a continuous basis. Our approach performs a comparison between the current person models and new unlabeled data to dynamically expand the gallery with new identities. We process the incoming information to maintain a small representative model of each person by exploiting concepts of information theory. The uncertainty and diversity of the new samples are analyzed to define which ones should be incorporated into the gallery. Experimental evaluation in challenging benchmarks includes an ablation study of the proposed framework, the assessment of different data selection algorithms that demonstrate the benefits of our approach, and a comparative analysis of the obtained results with other unsupervised and semi-supervised re-id methods.

## 1. Introduction

Person re-identification, or simply re-id, addresses the problem of matching people across non-overlapping views in a multi-camera system [1,2]. Solutions to this problem benefit many robotic applications where people are involved, such as tracking [3,4], navigation [5] or searching [6,7]. An extensive number of studies have focused on obtaining the best feature representation in supervised close-world scenarios (e.g., [8,9,10,11]) where the problem is narrowed to seek a query person from an existing pool of labeled people images, generally called *gallery*. While they obtain high performance in commonly used benchmarks, from the viewpoint of practical re-id systems, people identity annotation to obtain sufficient ground truth data could be extremely inefficient [12]. Hence, there is a tendency in the research community to address other alternatives and still open problems in re-identification, such as unsupervised [13,14,15], domain adaptation [16,17,18] or open-set in open-world [19,20,21]. The vast majority of these works use a static and preset gallery in their development that restrains the dynamic nature of the open-world, where raw data from camera systems collect new people, detection errors, or junk data. In order to solve problems related to open-world recognition, the system needs to deal with unknown classes but also be able to incrementally self-adapt by acquiring new knowledge [22,23]. Therefore, an open-world re-identification system should automatically evolve its gallery, be able to identify new identities and update known people’s data. To the best of our knowledge, existing approaches in person re-identification have not yet considered this fundamental problem of building a self-adaptive gallery. Thus, the lack of methods that address this problem motivates our research to propose a re-identification framework that focuses on the applicability of re-id approaches in open-world settings without any human assistance.

**Figure 1:**
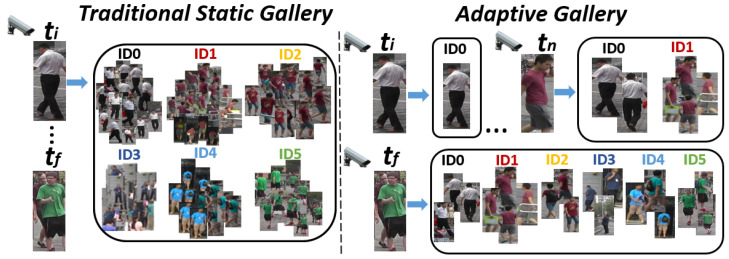
Simplified comparison between a large static gallery, traditionally used, and our small self-adaptive gallery. Both have a set of images representing each identity (ID0, ID1, …), i.e., each person. The traditional gallery is the same for every person query that arrives at different times ($t_{i}$, $t_{f}$). However, because the adaptive gallery is being built and updated as new data arrives, we can appreciate a more comprehensive gallery for later times ($t_{i}<t_{n}<t_{f}$).

This work presents a novel framework for person re-identification focusing on a self-adaptive gallery that evolves over time in an unsupervised fashion. The presented framework is able to dynamically expand to identify new individuals and build their appearance models with representative information. Figure 1 gives an overview of the differences between a labeled and static gallery traditionally used and our proposed adaptive gallery. Unlike the static gallery, we start with an empty gallery and update its structure as new samples arrive (unlabeled person images) to acquire new knowledge. The samples that provide the most representative appearance description of each person are selected to be included in the gallery. This selection is fully unsupervised and assembled using concepts of active learning techniques. Specifically, we analyze the uncertainty and diversity of each sample to evaluate its informativeness, keeping only those that present a good balance between low uncertainty and high diversity (less likely to be failures but not redundant with the rest). The main contributions of this work are: (1) A novel approach to build a self-adaptive gallery for person re-identification in open-world scenarios. The appearance model of each person is kept small and representative by selecting those samples that are most representative using information theory concepts. (2) A thorough evaluation of the posed problem. We include a metric based on the standard precision and recall to evaluate the quality of the gallery structure. This metric provides an intuition of the final quality of the gallery structure when the problem is complex and identifying the total number of classes is highly challenging.

The Section 4 provides a detailed analysis of the main parameters defined in the method, along with a comparison of different data selection algorithms commonly used in incremental settings. A comparison with other unsupervised and semi-supervised re-id methods is also discussed.

The rest of the paper is organized as follows. Section 2 details the related work. Section 3 describes the problem addressed, along with the main stages of the proposed framework. Section 4 presents a complete evaluation of the presented method on two challenging benchmarks. The first subsection analyzes the influence of the key parameter defined in the algorithm. Then, a comparison of different data selection methods demonstrates the benefits of our approach, and a discussion compares the proposed method with traditional approaches to re-identification. Finally, Section 5 concludes the work.

## 2. Related Work

The problem of person re-identification has been widely studied through time, as shown in [24]. Early works defined the problem as tracking [25], then moved to image-based classification [26] and video-based classification [27]. With the success of deep learning, works have shifted from hand-crafted descriptors [28] to deep learning methods [29]. The next step in person re-identification research was the shift from close-world (complete known classes and correctly annotated data) to open-world (multiple modalities, limited and noisy annotations, an undefined number of people, etc.) and has raised interesting new research challenges [22] relating the problem to other fields.

### 2.1. Unsupervised and Semi-Supervised Re-Id Methods

Several works attempt to tackle the re-id problem by building the re-id models in an unsupervised or semi-supervised manner. For example, Panda et al. [30] present a method to add a new camera to a multi-camera re-id system using unsupervised transfer learning from the knowledge obtained on the other cameras. Unsupervised algorithms typically focus on modeling the spatiotemporal information to match the people images between them [14,31], generate new data from unlabeled samples [32,33], or reduce the error in hard pseudo-labels using softer adaptable pseudo-labels [15]. Semi-supervised methods leverage the available annotated information by gradually refining the descriptors with the unlabeled data most similar to the labeled one [34] or by generating virtual samples based on the annotated data [35]. Different from these, we propose a method that focuses on creating a gallery that incrementally adds new unsupervised data, and we do not retrain the feature descriptors.

### 2.2. Incremental Person Re-Id

Incremental person re-identification has been approached from two main perspectives. First, the incremental adaptation of the learned model as new data arrives at the system [36]. This perspective trains the model in the same domain as the queries that will be analyzed later and uses a human in the loop to label the most representative data for the model adaptation through active learning techniques. Second, instead of adapting the feature representation, the goal is to perform a re-ranking in the gallery as new queries are matched with the labeled images [37]. Both perspectives use a static large gallery that ensures a match for the query person.

### 2.3. Gallery Construction

The construction of the gallery is based on the principle that instances of the same class are close in the feature space. This problem is often solved using clustering algorithms [31], which have been studied thoroughly in the literature [38,39] and applied in many fields. Close to our approach, DeCann et al. [40] present a work that updates the reference database (gallery) if the new data is not similar to any user by adding new users. However, they focus on different multi-modal information (face and finger) and an unlimited amount of data stored. To deal with the gallery construction problem in incremental scenarios, the available system resources should be taken into account since storing all the information received in a limitless fashion is not feasible. Therefore, the imposition of a bounded memory is commonly applied in many of these approaches [41,42]. Some works address the dynamical expansion of the classes aided by the labeling of the novel samples [43,44], while others also consider receiving new instances of already known classes, facing the challenges related to the update of existing class models [45,46]. They perform the update of each class model using a scoring system and controlling the size limit of each class by merging the most similar elements. This scenario is the most similar to our approach, but different from these existing works, our approach updates the model by analyzing not only the diversity of the samples but also the global uncertainty of the gallery. The result sought by combining both properties, obtaining a more varied model, is similar to that of prior work [47], which selects data with different levels of uncertainty from a set of labeled images. Different from all these methods, our approach deals with incremental and unlabeled information in an open-world scenario.

## 3. Method

This section describes in detail the addressed problem, the method overview, and the main stages of the proposed system.

### 3.1. Problem Description

We define the gallery as a set of classes, $𝓒=\{{𝓒}_{1},\ldots ,{𝓒}_{N}\},$ where each class, ${𝓒}_{i}\in 𝓒$, represents one person. Each class is represented by a set of at most *m* features ${𝓒}_{i}=\left\{f_{i}^{1},\ldots ,f_{i}^{m}\right\}$ with $f_{i}^{j}$ the *j*th feature of the class, respectively. The features are extracted from sample images, named samples for simplicity, and comprise an appearance descriptor, obtained from a generic re-id neural network, $x_{i}^{j}$, and the skeleton joints visible in the sample, $s_{i}^{j}$, $f_{i}^{j}=\left(x_{i}^{j},s_{i}^{j}\right)$. Specifically in this work, we select the re-identification Osnet model [9] to extract the appearance descriptors, and the OpenPose network [48] to obtain the skeleton joints.

The problem is to devise a method able to incrementally create the gallery from an empty initialization as new samples arrive in the system, considering an unknown (possibly unlimited) number of classes, *N*.

### 3.2. Method Overview

**Figure 2:**
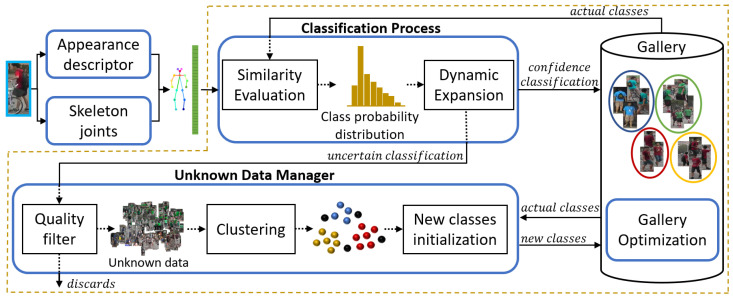
Self-adaptive gallery construction method overview. The person bounding box undergoes a pre-processing where the sample features are obtained with existing deep neural network encoders. Then, the proposed method analyzes the features obtained to decide which ones are used to adapt and evolve the gallery with the new information.

The overall idea of the proposed method is represented in Figure 2. First, whenever a new sample is acquired, the associated feature, $f_{q},$ is obtained. Then, the method performs a classification by computing the class probability distribution of the new sample through a similarity evaluation. Based on the confidence of the classification, the system decides whether to conduct a dynamic expansion or not. Samples with high confidence enter the gallery, while samples with low confidence are sent to the unknown data manager for further analysis. The set of unknown data is periodically clustered to generate new potential classes that are compared with the existing ones to identify and initialize new classes. Finally, since there is a limit in the memory budget of *m* features per class, the gallery optimization handles the efficient use of memory resources by deciding the relevant data to keep.

### 3.3. Classification Process

#### 3.3.1. Initialization Stage

In the initial phase of the gallery construction, the low number of classes initialized does not allow to work properly with probability distributions in the general regime. Therefore, the proposed system runs a short initialization stage. In order to perform this initialization, following the incremental setup, a set of candidate-classes, $𝔅=\{{𝔅}_{1},\ldots ,{𝔅}_{k}\},$ is defined, where the first candidate-class is created with the arrival of the first sample ${𝔅}_{1}=\left\{f_{1}^{1}\right\}$. Then, the similarity of incoming samples is evaluated by computing the cosine similarity between $x_{q}$ and those appearance descriptors already included in 𝔅. If the maximum cosine similarity is greater than a threshold, ε, the sample is included in the corresponding candidate-class set; otherwise, a new candidate-class is initialized. As soon as a candidate-class reaches a minimum size of *l*, it becomes a person-class, i.e., a real class, belonging to the gallery $𝓒=\{{𝓒}_{1}\}$. Once the gallery reaches a minimum number of person-classes, *Q*, the proposed decision-making based on the class probabilistic distribution of the samples is run as detailed next.

#### 3.3.2. General Regime

Once the gallery is initialized, the system **evaluates the similarity** of each new sample with the current gallery to obtain a probability distribution over the set of existing classes. This is accomplished using the softmax operator

(1) $$p\left(x_{q}\in {𝓒}_{i}\right)\equiv p_{i}\left(x_{q}\right)=\frac{\exp({\bar{x}}_{i}^{⊤}x_{q}/\upsilon )}{\sum_{j=1}^{N}\exp\left({\bar{x}}_{j}^{⊤}x_{q}/\upsilon \right)},$$

where υ is a temperature parameter that controls the softness of probability distribution over classes [31], $x_{q}$ is the normalized appearance descriptor of the new sample, and ${\bar{x}}_{i}$ is the weighted centroid of ${𝓒}_{i}$. Working with normalized vectors, the product of both descriptors, ${\bar{x}}_{i}^{⊤}x_{q}$, is equivalent to the cosine similarity between them. In this work, the weighted centroid ${\bar{x}}_{i}$ is defined as

(2) $${\bar{x}}_{i}=\frac{\sum_{j=1}^{m}r_{i}^{j}x_{i}^{j}}{\sum_{j=1}^{m}r_{i}^{j}},$$

$r_{i}^{j}=s_{i}^{j}/s_{T}$ being the ratio of joints visible in the person image bounding box with $s_{i}^{j}$ the number of detected joints and $s_{T}$ the total number of joints in a complete skeleton. Weighting the samples according to the number of joints favors the selection of samples with more body parts shown.

In a similar fashion to existing techniques for incremental learning [23,49], a threshold is used to control the **dynamic expansion** of the classes identified in the current gallery. More concretely, a simple and intuitive condition is used to measure the classification confidence of $x_{q}$ through its class probability distribution,

(3) $$\frac{\max_{i}p_{i}\left(x_{q}\right)}{\max_{j\neq i}p_{j}\left(x_{q}\right)}\geq \tau ,$$

where τ is the expansion threshold. Samples whose probability distribution does not comply with the condition (3) are considered doubtful and go into the pool of unknown data. Conversely, if the confidence of the classification obtained with (1) is higher or equal than τ, the pseudo-label assigned to the sample corresponds to the class with maximum probability, $i^{*}=\arg\max_{i}p_{i}\left(x_{q}\right)$, and will be considered to be part of its representation model, ${𝓒}_{i^{*}}$.

### 3.4. Unknown Data Manager

Samples that do not satisfy the classification confidence criteria (3) are defined as unknown. The role of the Unknown Data Manager is to identify new identities as well as to recover samples that could not be previously classified with enough certainty. To avoid the initialization of new classes with sets of poorly-explained features, i.e., images showing only one arm or one leg, all the unknown samples first undergo a quality filter to ensure that the appearance descriptors represent at least half of a person, formally r≥0.5, *r* being the ratio of joints.

The identification of new classes is tackled through the periodic **clustering** of the unknown data. In open-world scenarios, the number of classes is unbounded, making the use of clustering methods such as K-Means unfeasible. Thus, to partition the set of unknown data, we use a DBSCAN algorithm [50] based on sample density and can deal with noisy information. The resulting clusters that reach the minimum size of *l* are compared with the current classes in the gallery to check whether they belong to an existing class or represent a new one. Following the analysis performed in [31] on criteria methods to decide which pair of clusters to merge, the minimum distance criterion is used to verify if a potential new class, ${𝓒}_{w}$, shares identity with any of the existing in the gallery. The minimum distance criterion takes the shortest distance between samples from the new cluster, ${𝓒}_{w}$, and all elements of the gallery, 𝓒,

(4) $$D\left({𝓒}_{w},𝓒\right)=\min_{{𝓒}_{i}\in 𝓒}\left(\min_{x_{j}\in {𝓒}_{i},x\in {𝓒}_{w}}\left(1-x^{⊤}x_{j}\right)\right).$$

Since the computational cost of this process is considerably high, we compute an approximation limiting the number of existing classes that are compared with ${𝓒}_{w}$ from the set *N* to a subset of *k*. To select which classes are analyzed, for each $x\in {𝓒}_{w},$ we compute the *k*-Nearest centroids of the gallery and then select the *k* most frequent classes among all of them. Using only these classes in the first minimum of (4), the computational cost remains constant with the size of the gallery.

Finally, if the approximated minimum distance is higher than α, the cluster ${𝓒}_{w}$ is **initialized in the gallery as a new class**. Otherwise, the new cluster and the class with the closest sample represent the same identity and are merged, complying with the memory budget by means of the gallery optimization process.

### 3.5. Gallery Optimization

Our approach performs an intelligent decision-making process with the goal of storing representative features of each existing class and making efficient use of memory resources. In order to address this goal, we use two metrics that describe the relationship of each appearance descriptor with those in the same class and with all the rest.

The **first metric is the intra-class diversity** of the samples. For a descriptor, *x*, that belongs to class ${𝓒}_{i}$, we define its diversity through the minimum cosine distance among all the other descriptors that belong to the same class,

(5) $$D_{i}\left(x\right)=\min_{x_{j}\in {𝓒}_{i}\backslash x}\left(1-x^{T}x_{j}\right).$$

The diversity of the whole class is then defined as the minimum diversity among all of its features,

(6) $$D\left({𝓒}_{i}\right)=\min_{x_{j},x_{k}\in {𝓒}_{i},x_{j}\neq x_{k}}\left(1-x_{j}^{T}x_{k}\right).$$

This metric is useful to identify redundant information, i.e., similar samples within a class. Leveraging this information, when a new sample is classified and assigned to an existing class of the gallery, ${𝓒}_{i}$, it is only added to the representation model of the class if its diversity is greater than the current diversity of the class,

(7) $$D_{i}\left(x_{q}\right)\geq D\left({𝓒}_{i}\right).$$

The **second metric is the uncertainty** of the sample with respect to the whole gallery, which is measured through Shannon’s entropy by

(8) $$H\left(x\right)=-\sum_{i=1}^{N}p_{i}\left(x\right)\log\left(p_{i}\left(x\right)\right),$$

where *N* is the number of classes at the moment in the gallery, and $p_{i}\left(x\right)$ is the probability described in (1). High entropy values stand for appearance descriptors that can be easily confused with those of other classes. In contrast, a feature with low entropy indicates high confidence in belonging to a certain class. Therefore, this metric provides an intuition of the relative distance between the feature and the rest of the classes of the gallery (inter-class).

The dependency on all the classes in (8), together with the constant evolution of the class centroids required for (1), makes the computation of this metric very heavy. For efficient computation, we keep a matrix for each class, $R_{i},$ with the cosine similarity between its samples, $x_{i}^{j}$, and all the weighted centroids of the gallery,

(9) $$R_{i}=\left[\begin{array}{cccc} {\bar{x}}_{1}^{⊤}x_{i}^{1} & {\bar{x}}_{2}^{⊤}x_{i}^{1} & \cdots & {\bar{x}}_{N}^{⊤}x_{i}^{1}\\ {\bar{x}}_{1}^{⊤}x_{i}^{2} & {\bar{x}}_{2}^{⊤}x_{i}^{2} & \cdots & {\bar{x}}_{N}^{⊤}x_{i}^{2}\\ \vdots & \vdots & \ddots & \vdots \\ {\bar{x}}_{1}^{⊤}x_{i}^{m} & {\bar{x}}_{2}^{⊤}x_{i}^{m} & \cdots & {\bar{x}}_{N}^{⊤}x_{i}^{m} \end{array}\right],$$

as well as a list of the classes that have changed since the last gallery optimization of ${𝓒}_{i}$ was performed. This list is used to update only the columns associated with classes with changes, noting that the other distances have not changed and can be reused. Note that the $R_{i}$ matrix is the changing element of (1) since υ is a constant value. Once we compute the update of the probability distribution of the samples belonging to ${𝓒}_{i}$, obtaining entropy with (8) is straightforward.

When the memory budget of a class is exceeded, because of a merge caused by the Unknown Data Manager or the insertion of a new sample, an optimization process using both metrics is run to decide which sample to drop. In particular, the sample to drop is

(10) $$x^{*}=\underset{x\in {𝓒}_{i}}{arg max}\left(\gamma \frac{H\left(x\right)}{\log\left(1/N\right)}-\left(1-\gamma \right)D_{i}\left(x\right)\right),$$

where γ∈[0,1] is a parameter to weigh the relevance of the uncertainty and the diversity terms. The logarithm, $\log\left(1/N\right)$, normalizes the entropy to a value between zero and one, equivalent to the diversity. The proposed optimization function seeks a balance between how much a given feature mixes the different classes (entropy) and how distinctive it is with respect to the rest of the features of the same class (diversity).

**Figure 3:**
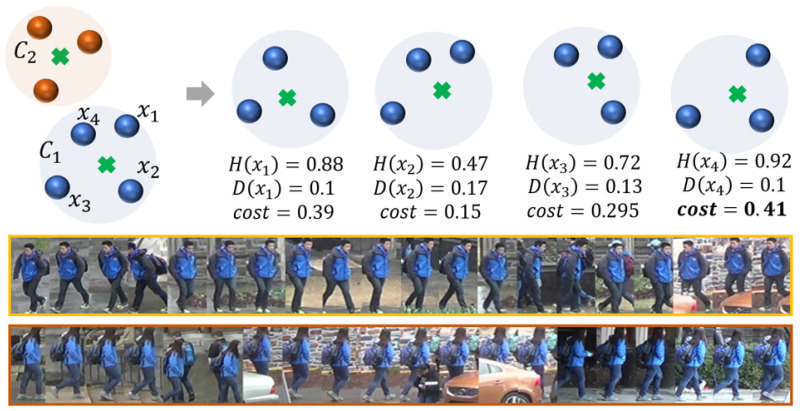
Gallery optimization. Upper area: example simplified where $C_{1}$ exceeds the memory budget and the gallery optimization selects the feature with the maximum cost to be dropped, $x_{4}$. Lower area: visual sample of two appearance models from similar identities that are correctly separated in the *DukeMTMC-VideoReID* dataset. The yellow edge corresponds to identity 86 and the orange edge to identity 194, both ground truth identities.

Figure 3 shows a simplified example with two clusters, ${𝓒}_{1}$ and ${𝓒}_{2}$, where ${𝓒}_{1}$ has exceeded its size constraint m=3, and two examples of the final appearance models obtained with the proposed process. Note the balance between uncertainty and diversity even though the two identities look very similar.

## 4. Experiments

This section analyzes the influence of the main parameters defined in the system, the algorithm selected to model the person’s appearance and compares the performance of the proposed framework with other unsupervised and semi-supervised re-id approaches.

### 4.1. Experimental Setup

The evaluation is performed with two public benchmarks, MARS [51] and DukeMTMC-VideoReID [34]. In both of them, we use the official test set, which is split into the *query set* and the *gallery set*.

Two experiments are performed in this section. First, the analysis of the *gallery construction* process assesses the key aspects of our approach. The second experiment, *query re-identification*, runs a conventional evaluation for re-id methods in order to compare the proposed framework with other unsupervised and semi-supervised approaches. For both experiments, the settings for our approach configuration are: similarity threshold in the initialization stage ε=0.9, temperature parameter in the softness operator υ=0.1, the *k*-Nearest centroids with k=3 used by the Unknown Data Manager, distance threshold to initialize a new cluster α=0.1, gallery size to run the probabilistic decision making Q=20, the re-identification network used in cross-domain is an OsNet model [9] trained with the MSMT17 Benchmark [52], and the OpenPose network [48] is used to obtain the skeleton joints. The setup for both experiments is detailed next.

#### 4.1.1. Gallery Construction

The *gallery set* from both datasets is used to evaluate the self-adaptive gallery construction process. As in traditional incremental settings, the tracklets are randomly shuffled, and then, the images from each tracklet are provided one by one to simulate an incremental input to the self-adaptive gallery.

In order to evaluate the global performance of the proposed approach, we consider the following three metrics based on the classic precision, recall, and F1 score:

- **Gallery Structure**: The perfect gallery structure has one (and only one) class per ground truth identity (GT-ID). This GT-ID is set for each class with the mode of all the sample identities present at the class initialization. In order to evaluate the quality of the final gallery structure, we compute the **precision (P)**, **recall (R)**, and **F1 score** metrics as
  (11) $$\begin{array}{c} P=\frac{TP}{TP+FP},\;R=\frac{TP}{TP+FN}\;\;\;\mathrm{and}\;\;\;F1=\frac{2\;\cdot \;(P\;\cdot \;R)}{P+R}, \end{array}$$
  where we define the false negatives (FN) as those GT-ID not associated with any class, i.e., identities not found, the true positives (TP) as all GT-IDs associated with at least one class, i.e., identities found, and the false positives (FP) as the additional classes with the same GT-ID associated, i.e, two classes associated to the same GT-ID count as one FP and one TP.
- **Class Precision**: This metric assesses the precision of the samples that enter the gallery over time. The true positives (TP) are the samples whose identity matches the GT-ID of the class they have been assigned, and the false positives (FP) are the samples that do not.
- **Sample Classification F1**: This metric evaluates the pseudo-label assigned to every sample that arrives to the system. Considering that the gallery structure often has redundancy due to the unsupervised nature of the system, we deem a limited number of redundant classes for each identity. In particular, for a given GT-ID, we only consider the *K* classes with the highest number of samples associated with them, discarding the rest. The true positives (TP) are the samples that match the GT-ID with the assigned class. The false positives (FP) are the samples with mismatching GT-IDs, and the false negatives (FN) are samples classified as unknown or assigned to the discarded classes.

#### 4.1.2. Query Re-Identification

In order to compare the proposed framework with other unsupervised and semi-supervised approaches, we use the *query set* to evaluate the gallery obtained at the end of the *gallery construction* process. Thus, the *query set* is matched with the limited size gallery created in the previous experiment, which remains static during this evaluation. The conventional evaluation for re-identification [9] is performed including the **Rank-1** and **Rank-5** metrics.

### 4.2. Gallery Construction: Parameter Evaluation

We first study the effect of the three key parameters for the gallery construction process: (1) the weight used in Equation (10) to balance the influence of the uncertainty and the diversity, γ, (2) the expansion threshold, τ, in Equation (3), and (3) the minimum size required to initialize a class, *l*, used during the initialization stage and the clustering process, along with the memory budget per identity, *m*, defined in Section 3.1. In this evaluation, we use K=4 for the *sample classification F*1. The goal of this analysis is to choose the parameters that yield balanced galleries based on the defined metrics.

**Figure 4:**
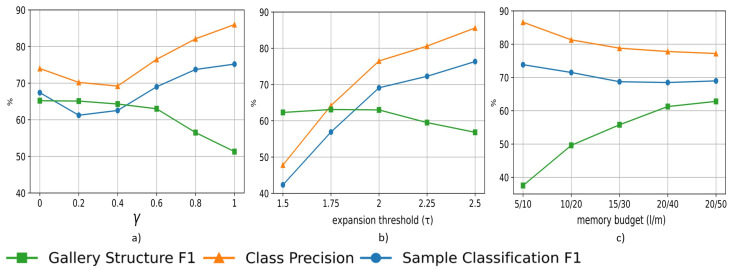
Parameter evaluation in the gallery construction process using the MARS dataset: (**a**) effect of the weight assigned to uncertainty and diversity (γ); (**b**) influence of the expansion threshold (τ); (**c**) effect of the minimum size to create a class and the memory budget (l/m).

The results of the analysis are shown in Figure 4. The influence of each parameter at the end of the process is analyzed in Figure 4a–c, where it can be seen that the trend of the quality *gallery structure F*1 is inverse to the tendency of the *class precision* and the *sample classification F*1.

Figure 4a shows the effect of **weighting the uncertainty and diversity** with γ, fixing all the other parameters to τ=2, l=20 and m=50. The increase in γ favors the selection of samples with low entropy but less diverse ones in the appearance models. The balance between uncertainty and diversity in the gallery is attained at γ=0.6.

The **expansion threshold**, τ, is analyzed in Figure 4b. We keep l=20, m=50, and from the former analysis, γ is set to 0.6. When this parameter increases, more samples are sent to the Unknown Data Manager, resulting in the initialization of more classes. The trade-off between the metrics analyzed is accomplished at τ=2.

Finally, the influence of the minimum size to create a class, *l*, and the **memory budget** per identity, *m*, is evaluated in Figure 4c. The rest of the parameters are set to γ=0.6, τ=2. The increase in the *gallery structure F*1 is caused by the reduction in the initialization, leading to fewer redundant classes. This implies greater confidence in the classification of the samples as *m* increases. Therefore, the selected memory budget configuration is the one that generates the highest *gallery structure F*1, l=20 and m=50, the influence being not highly significant in the other metrics analyzed.

**Figure 5:**
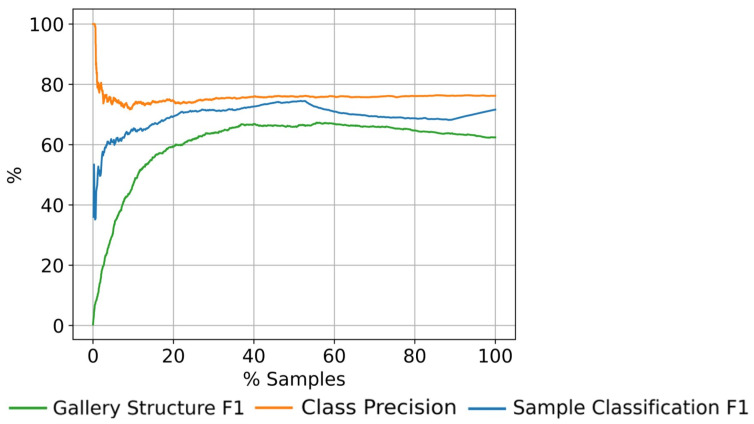
Evolution over time of the metrics with the final parameters set in the gallery construction process using the MARS dataset .

Figure 5 shows the evolution over time of the metrics with the final parameters set, γ=0.6, τ=2, l=20, and m=50. Since it is an evaluation over time, in this particular case we consider K=∞ for the *sample classification F*1. All the metrics settle after processing 20% of the samples. Then, it can be fairly assumed that the method’s behavior is stable beyond that stage.

### 4.3. Gallery Construction: Data Selection Method Comparison

Following the analysis from the previous section, this experiment sets γ=0.6, τ=2, l=20, and m=50. We study different gallery optimization processes that decide which sample to remove from the appearance model when the memory budget is exceeded. The compared techniques are algorithms used in incremental clustering works that have to deal with memory budget requirements. They are evaluated at the end of the gallery construction process. The first method is uniform sampling (Uniform) which saves a new feature for every U=5 instance. When the size limit is exceeded, the oldest data is dropped to save a newer one. Another typical process is random decision-making (Random) which removes a random index when the memory reaches its budget. Regarding more sophisticated methods, we compare the two closest approaches in the literature, the method proposed in [45], called Incremental Object Model (IOM), and the ExStream method [46]. In both cases, we use the implementation provided by the authors to evaluate the effect of the data dropped in the gallery in our overall method. Moreover, due to the influence on the final results of the data arrival order in incremental setups, three different iterations are run (i.e., three different random data arrival orders). To make a fair comparison, all five methods use the same features extracted from OsNet [9].

**Figure 6:**
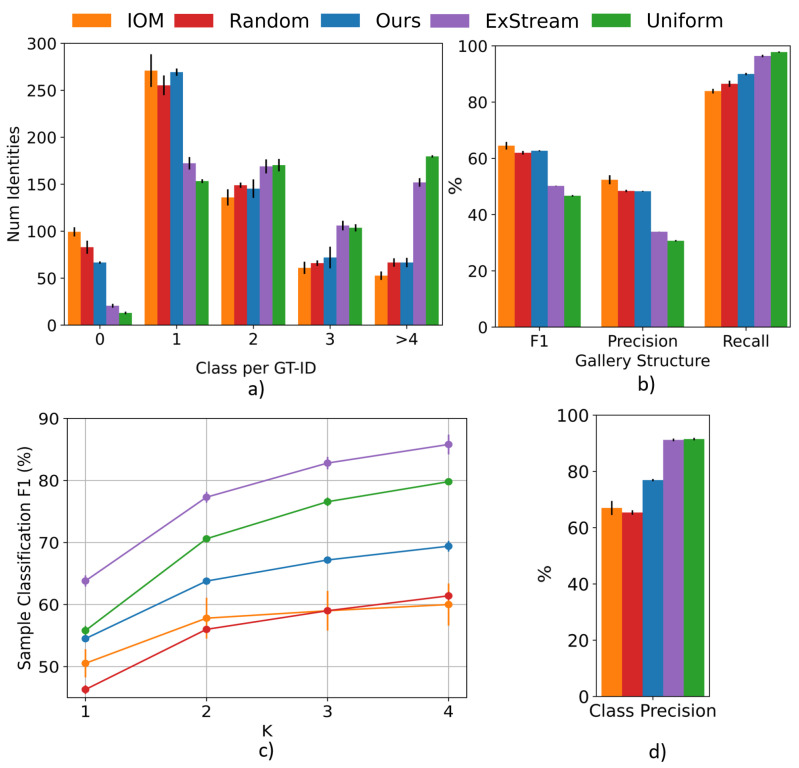
Data selection method comparison with the MARS dataset. We analyze (**a**) the number of classes created (*x*-axis) per GT-ID in the dataset (*y*-axis) showing the number of GT-ID with more than one class associated or those GT-ID that have not been correctly found, i.e., 0 classes associated; (**b**) *gallery structure* metrics: F1, precision, and recall; (**c**) *sample classification F1* analyzing the influence of varying *K*; (**d**) *class precision*.

First, a comprehensive analysis of the final quality of the *gallery structure* is performed. The number of classes created per GT-ID and the *gallery structure* metrics are shown in Figure 6a,b, respectively. The results in Figure 6a indicate that the ExStream and the Uniform algorithms create a high number of redundant classes in the gallery. This means that the appearance models resulting from these methods are significantly less representative, leading to more uncertain classifications. Thus, they send a high number of samples to the unknown pool and create new classes for already existing identities. The proposed optimization process (Ours) creates only one class for the same number of GT-IDs as IOM while identifying more people in the scene, which is represented by a smaller number of GT-IDs with 0 classes created. Then, derived from this analysis and verified in the F1 results on Figure 6b, the methods which provide a gallery structure of better quality are IOM, Random, and Ours, being Ours the one that identifies the most people in the scene among them, as measured with the *gallery structure recall*.

Second, Figure 6c shows the analysis of varying *K* in the *sample classification F1*, and Figure 6d shows the *class precision* results. As expected, the *sample classification F1* improves in all algorithms with the increment of *K*. Comparing the methods that generate a gallery with a suitable structure, i.e., IOM, Random, and Ours, the results shown in Figure 6c,d demonstrate that the proposed gallery optimization process (Ours) outperforms IOM and Random in both metrics. Our approach is able to create more reliable people models without losing diversity, thus enhancing the classification of the samples. The ExStream and Uniform methods obtain high values in these metrics because of the large number of redundant classes, limiting in practice the actual ability to re-identify known people.

As a summary of the experiment, our algorithm is the one that maintains the best balance between having a good *gallery structure* and providing good classification metrics of the individual samples with it. The rest of the methods either generate galleries with worse quality structure, i.e., ExStream and Uniform, or obtain worse *class precision* and *sample classification F*1 results, i.e., IOM and Random.

### 4.4. Gallery Construction: Final Results

A detailed evaluation on MARS and DukeMTMC-VideoReID is provided using the same hyperparameter values from the previous section for both benchmarks.

Table 1 shows the final results of the complete self-adaptive gallery construction approach on both datasets. In the *gallery structure* analysis, the table includes the number of GT-IDs, classes created and the gallery structure F1, the precision, and the recall. The larger number of people in DukeMTMC-VideoReID makes it more challenging to identify most of them, causing lower recall metrics than in the MARS dataset, i.e., the 80.06% of the people have been correctly identified in DukeMTMC-VideoReID against the 89.43% in MARS (*gallery structure recall*). In terms of *class precision*, note that the proposed framework obtains similar and consistent results for both datasets, 76.69% in MARS and 80.1% in DukeMTMC-VideoReID. Thus, the method creates robust appearance models, being able to correctly distinguish the people in the scene, which in turn helps in the *sample classification* obtaining precision results of 72%.

**Figure 7:**
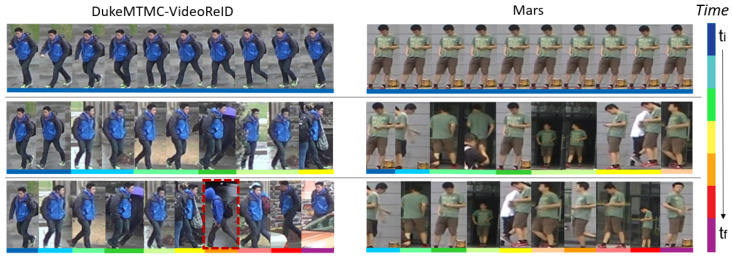
Visualization of the evolution of appearance models in the gallery. Each row corresponds to gallery samples at a certain time. The different colors represent the time stamp of the samples included in the gallery (best viewed in color).

Finally, Figure 7 includes samples of the gallery for one identity per dataset at three different times during their construction, showing in each row the person model at different times. The left identity includes an example of corruption that the gallery can suffer remarked by a discontinuous red line.

In both cases, the third row shows how our resulting gallery presents high variability of samples, resulting in a representative model for each identity. More detailed qualitative results of the proposed self-adaptive gallery can be seen in the Supplementary Material, where the identification of new classes and the evolution of the people’s appearance models are shown.

### 4.5. Query Re-Identification

This final experiment performs the traditional evaluation of person re-id, i.e., obtains the expectation that the true match is found within the first *R* ranks [53]. However, instead of matching the *query set* with a completely labeled gallery, the *query set* is matched with the resulting gallery from the *gallery construction* process. In this experiment, the gallery remains static. The proposed method obtains its results in an incremental unsupervised cross-domain setting (IUCD). Table 2 shows the results of this experiment, including the setting in which the different methods operate. Our offline baseline is the Full-gallery method, which has the whole gallery available and manually labeled using the same descriptors as our approach. This method is our upper bound result in the cross-domain setting. Moreover, due to the unsupervised component of our approach, we present the results of unsupervised and semi-supervised systems that perform offline training in the same domain as the *query set*. The unsupervised methods that included BUC [31], softened sim [15] and GLC+ [32] do not use any labeled data in the whole process (None). Concerning the semi-supervised approaches, they use one tracklet labeled per identity (OneEx). Note that we are the only algorithm working on the incremental unsupervised cross-domain (IUCD) setting, while the rest perform the entire process offline. Thus, although Table 2 is not a fair or direct comparison for our approach, we believe that it is interesting to see how close the proposed approach results are with respect to existing methods, despite the much more challenging and realistic scenario of our approach. The resulting values for our approach are the average and the standard deviation for the three random iterations performed previously, i.e., mean (±std). Besides, since the proposed gallery deals with memory requirements, the percentage of the gallery size used with respect to the total (GS) is shown. In this case, the standard deviation is not included, but we remark that it is lower than 0.01 in all cases.

The DukeMTMC-VideoREID results show the impact of the different goals sought. In our case, the correct identification of the 1110 people that compose the gallery is a really challenging task, where some of the queries analyzed in this evaluation do not have corresponding models in the gallery. In contrast, the methods that focus on improving the feature representation obtain better results than in the MARS dataset due to the lack of distractors in the gallery. Regarding the MARS dataset, which is closer to an open-world scenario, the results with our approach are close to the unsupervised or semi-supervised approaches using two orders of magnitude less in the amount of data stored in the gallery. Finally, considering the difference between the Full-gallery baseline and our approach, we see how the proposed approach achieves comparable performance despite a much smaller (one or two orders of magnitude less) and unsupervised built gallery.

## 5. Conclusions

This work has presented a novel framework to address the problem of person re-id in open-world able to detect new identities and update the model about existing identities in the system. To deploy and evaluate intelligent systems in open-world settings, it is essential to be able to bridge certain gaps, such as lack of supervised data or lack of computational resources. In particular, the proposed approach shows how to build a self-adaptive gallery for person re-identification in a fully unsupervised fashion, while managing limited memory resources. Low supervision and resource requirements are key to robotics applications in the real world, so our self-adaptive gallery can boost robotic tasks that involve people in real-world applications, such as information gathering or searching. The main limitations of the presented work are those inherent to the re-identification systems, concerning long-term person re-id when there is a change of clothing or strong appearance changes in the people being monitored. In this situation, our system is likely to start a new class under the assumption that a new identity has appeared on the scene. Future steps to improve this aspect may include re-identification models focused on long-term robustness. In the short-term person re-identification problem, our framework can identify more than 80% of the people presented in the challenging scenarios evaluated by comparing the new unlabeled data and the existing classes in the gallery. The existing classes in the gallery are modeled with an optimization process that selects the most representative information to represent each class, balancing the uncertainty (inter-class) and the diversity (intra-class) of the samples. The experiments carried out demonstrate that the proposed optimization process returns a *class precision* of about 80% while encouraging the variability inside the classes, generating well-balanced and more structured galleries than those of the similar existing methods analyzed. The high *class precision* maintained over time aids the continuous person re-id by obtaining an *F1 sample classification* of 62.6% and 69.4% in the Mars and Duke datasets, respectively. Compared to existing re-id algorithms, our method obtains similar results to the fully labeled galleries storing one or two orders of magnitude less data.

## Figures and Tables

**Table 1:** Detailed results of the proposed framework on the MARS and DukeMTMC-VideoReID datasets. The results show the mean and the standard deviation of the three iterations performed, mean (±std).

Metrics	Dataset	
	MARS	DukeMTMC-VideoReID
***Gallery Structure***		
Total IDs (GT)	620	1110
Classes Created	1147.6 (±2.5)	1337.33 (±16)
F1	62.67 (±0.19)	72.62 (±0.26)
Precision	48.24 (±0.12)	66.45 (±0.51)
Recall	89.43 (±0.4)	80.06 (±0.47)
***Class Precision***	76.9 (±0.36)	80.1 (±0.60)
***Sample Classification***		
F1	69.4 (±0.86)	62.6 (±0.87)
Precision	72.23 (±0.12)	72.43 (±1.12)
Recall	66.8 (±1.69)	55.21 (±0.69)

**Table 2:** Comparison with re-id approaches in DukeMTMC-VideoReID and MARS *query set*.

Method	Setting	DukeMTMC-VideoReID			MARS		
		GS (%)	Rank-1	Rank-5	GS (%)	Rank-1	Rank-5
Full-gallery	Cross-Domain	100	63.2	72	100	66.4	73.3
EUG [34]	OneEx	100	72.7	84.1	100	62.67	74.94
SCLU [54]	OneEx	100	72.7	85	100	63.74	78.44
BUC [31]	Unsp. (None)	100	76.2	88.3	100	57.9	72.3
Softened Sim [15]	Unsp. (None)	100	76.4	88.7	100	62.7	77.2
GLC+ [32]	Unsp. (None)	100	80.9	91.5	100	66.5	78.7
Ours	IUCD	18.4	59.5 (±1.2)	69.1 (±1.04)	8.1	60.1 (±0.78)	69.8 (±0.38)

## Data Availability

The MARS people re-identification dataset is available at: http://zheng-lab.cecs.anu.edu.au/Project/project_mars.html (accessed on 18 January 2023). The DukeMTMC-VideoReID people re-identification dataset is available at: https://github.com/Yu-Wu/DukeMTMC-VideoReID (accessed on 18 January 2023).

## Supplementary Materials

The following supporting information can be downloaded at: https://unizares-my.sharepoint.com/:v:/g/personal/scasao_unizar_es/EZWQrO_Qk4ZNjaSdFT63AQIBEHD672uPJKKTLVi6OhcdXQ?e=maXZHo, Video: Supplementary.mp4.

## Abbreviations

The following abbreviations are used in this manuscript:

DBSCAN	Density-Based Spatial Clustering of Applications with Noise
MARS	Motion Analysis and Re-identification Set
GT-ID	Ground Truth Identity
IOM	Incremental Object Model
IUCD	Incremental Unsupervised Cross-Domain
BUC	Bottom-Up Clustering
EUG	Exploit Unknown Gradually
SCLU	one-Shot person re-identification with Collaboration between the
	Labeled and Unlabeled

## References

[1] Wu D., Zheng S.J., Zhang X.P., Yuan C.A., Cheng F., Zhao Y., Lin Y.J., Zhao Z.Q., Jiang Y.L., Huang D.S. (2019). Deep learning-based methods for person re-identification: A comprehensive review. Neurocomputing.

[2] Ye M., Shen J., Lin G., Xiang T., Shao L., Hoi S.C. (2021). Deep learning for person re-identification: A survey and outlook. IEEE Trans. Pattern Anal. Mach. Intell..

[3] De Langis K., Sattar J. Realtime multi-diver tracking and re-identification for underwater human-robot collaboration. Proceedings of the International Conference on Robotics and Automation.

[4] Chang F.M., Lian F.L., Chou C.C. (2015). Integration of modified inverse observation model and multiple hypothesis tracking for detecting and tracking humans. IEEE Trans. Autom. Sci. Eng..

[5] Truong X.T., Ngo T.D. (2017). Toward socially aware robot navigation in dynamic and crowded environments: A proactive social motion model. IEEE Trans. Autom. Sci. Eng..

[6] Shree V., Chao W.L., Campbell M. (2020). Interactive Natural Language-Based Person Search. IEEE Robot. Autom. Lett..

[7] Mohamed S.C., Rajaratnam S., Hong S.T., Nejat G. (2019). Person finding: An autonomous robot search method for finding multiple dynamic users in human-centered environments. IEEE Trans. Autom. Sci. Eng..

[8] Wen Z., Sun M., Li Y., Ying S., Peng Y. Asymmetric Local Metric Learning with PSD Constraint for Person Re-identification. Proceedings of the International Conference on Robotics and Automation.

[9] Zhou K., Yang Y., Cavallaro A., Xiang T. (2021). Learning generalisable omni-scale representations for person re-identification. Trans. Pattern Anal. Mach. Intell..

[10] Luo H., Jiang W., Zhang X., Fan X., Qian J., Zhang C. (2019). Alignedreid++: Dynamically matching local information for person re-identification. Pattern Recognit..

[11] Hou R., Ma B., Chang H., Gu X., Shan S., Chen X. (2021). Feature completion for occluded person re-identification. IEEE Trans. Pattern Anal. Mach. Intell..

[12] Leng Q., Ye M., Tian Q. (2019). A survey of open-world person re-identification. IEEE Trans. Circuits Syst. Video Technol..

[13] Wang D., Zhang S. Unsupervised person re-identification via multi-label classification. Proceedings of the Conference on Computer Vision and Pattern Recognition.

[14] Sridhar Raj S M.V.P., Balakrishnan R. (2022). Spatio-Temporal association rule based deep annotation-free clustering (STAR-DAC) for unsupervised person re-identification. Pattern Recognit..

[15] Lin Y., Xie L., Wu Y., Yan C., Tian Q. Unsupervised person re-identification via softened similarity learning. Proceedings of the Conference on Computer Vision and Pattern Recognition.

[16] Feng H., Chen M., Hu J., Shen D., Liu H., Cai D. (2021). Complementary pseudo labels for unsupervised domain adaptation on person re-identification. IEEE Trans. Image Process..

[17] Wang G., Lai J.H., Liang W., Wang G. Smoothing adversarial domain attack and p-memory reconsolidation for cross-domain person re-identification. Proceedings of the Conference on Computer Vision and Pattern Recognition.

[18] Zheng Y., Tang S., Teng G., Ge Y., Liu K., Qin J., Qi D., Chen D. Online pseudo label generation by hierarchical cluster dynamics for adaptive person re-identification. Proceedings of the International Conference on Computer Vision.

[19] Huang Y., Zha Z.J., Fu X., Hong R., Li L. Real-world person re-identification via degradation invariance learning. Proceedings of the Conference on Computer Vision and Pattern Recognition.

[20] Zhao Y., Li Y., Wang S. (2019). Open-world person re-identification with deep hash feature embedding. IEEE Signal Process. Lett..

[21] Martini M., Paolanti M., Frontoni E. (2020). Open-world person re-identification with rgbd camera in top-view configuration for retail applications. IEEE Access.

[22] Bendale A., Boult T. Towards open world recognition. Proceedings of the Conference on Computer Vision and Pattern Recognition.

[23] Fontanel D., Cermelli F., Mancini M., Bulo S.R., Ricci E., Caputo B. (2020). Boosting deep open world recognition by clustering. IEEE Robot. Autom. Lett..

[24] Zheng L., Yang Y., Hauptmann A.G. (2016). Person re-identification: Past, present and future. arXiv.

[25] Zajdel W., Zivkovic Z., Krose B.J. Keeping track of humans: Have I seen this person before?. Proceedings of the International Conference on Robotics and Automation.

[26] Gheissari N., Sebastian T.B., Hartley R. Person re-identification using spatio-temporal appearance. Proceedings of the Conference on Computer Vision and Pattern Recognition.

[27] Bazzani L., Cristani M., Perina A., Farenzena M., Murino V. Multiple-shot person re-identification by hpe signature. Proceedings of the International Conference on Pattern Recognition.

[28] Zhao R., Ouyang W., Wang X. Unsupervised salience learning for person re-identification. Proceedings of the Conference on Computer Vision and Pattern Recognition.

[29] Yi D., Lei Z., Liao S., Li S.Z. Deep metric learning for person re-identification. Proceedings of the International Conference on Pattern Recognition.

[30] Panda R., Bhuiyan A., Murino V., Roy-Chowdhury A.K. (2019). Adaptation of person re-identification models for on-boarding new camera(s). Pattern Recognit..

[31] Lin Y., Dong X., Zheng L., Yan Y., Yang Y. A bottom-up clustering approach to unsupervised person re-identification. Proceedings of the AAAI Conference on Artificial Intelligence.

[32] Chen H., Wang Y., Lagadec B., Dantcheva A., Bremond F. (2022). Learning Invariance from Generated Variance for Unsupervised Person Re-identification. arXiv.

[33] Zhang X., Li D., Wang Z., Wang J., Ding E., Shi J.Q., Zhang Z., Wang J. Implicit sample extension for unsupervised person re-identification. Proceedings of the Conference on Computer Vision and Pattern Recognition.

[34] Wu Y., Lin Y., Dong X., Yan Y., Ouyang W., Yang Y. Exploit the unknown gradually: One-shot video-based person re-identification by stepwise learning. Proceedings of the Conference on Computer Vision and Pattern Recognition.

[35] Han H., Zhou M., Shang X., Cao W., Abusorrah A. (2020). KISS+ for rapid and accurate pedestrian re-identification. IEEE Trans. Intell. Transp. Syst..

[36] Martinel N., Das A., Micheloni C., Roy-Chowdhury A.K. Temporal model adaptation for person re-identification. Proceedings of the European Conference on Computer Vision.

[37] Wang Z., Jiang J., Yu Y., Satoh S. (2019). Incremental re-identification by cross-direction and cross-ranking adaption. IEEE Trans. Multimed..

[38] Xu R., Wunsch D. (2005). Survey of clustering algorithms. IEEE Trans. Neural Netw..

[39] Xu D., Tian Y. (2015). A comprehensive survey of clustering algorithms. Ann. Data Sci..

[40] DeCann B., Ross A. (2015). Modelling errors in a biometric re-identification system. IET Biom..

[41] Rebuffi S.A., Kolesnikov A., Sperl G., Lampert C.H. icarl: Incremental classifier and representation learning. Proceedings of the Conference on Computer Vision and Pattern Recognition.

[42] Belouadah E., Popescu A. IL2M: Class incremental learning with dual memory. Proceedings of the International Conference on Computer Vision.

[43] Mancini M., Karaoguz H., Ricci E., Jensfelt P., Caputo B. Knowledge is never enough: Towards web aided deep open world recognition. Proceedings of the International Conference on Robotics and Automation.

[44] Valipour S., Perez C., Jagersand M. Incremental learning for robot perception through HRI. Proceedings of the International Conference on Intelligent Robots and Systems.

[45] Azagra P., Civera J., Murillo A.C. (2020). Incremental Learning of Object Models From Natural Human–Robot Interactions. IEEE Trans. Autom. Sci. Eng..

[46] Hayes T.L., Cahill N.D., Kanan C. Memory efficient experience replay for streaming learning. Proceedings of the International Conference on Robotics and Automation.

[47] Bang J., Kim H., Yoo Y., Ha J.W., Choi J. Rainbow Memory: Continual Learning with a Memory of Diverse Samples. Proceedings of the Conference on Computer Vision and Pattern Recognition.

[48] Cao Z., Hidalgo Martinez G., Simon T., Wei S., Sheikh Y.A. (2019). OpenPose: Realtime Multi-Person 2D Pose Estimation using Part Affinity Fields. arXiv.

[49] Rao D., Visin F., Rusu A., Pascanu R., Teh Y.W., Hadsell R. (2019). Continual Unsupervised Representation Learning. Adv. Neural Inf. Process. Syst..

[50] Ester M., Kriegel H.P., Sander J., Xu X. A density-based algorithm for discovering clusters in large spatial databases with noise. Proceedings of the KDD.

[51] Zheng L., Bie Z., Sun Y., Wang J., Su C., Wang S., Tian Q. Mars: A video benchmark for large-scale person re-identification. Proceedings of the European Conference on Computer Vision.

[52] Wei L., Zhang S., Gao W., Tian Q. Person transfer gan to bridge domain gap for person re-identification. Proceedings of the Conference on Computer Vision and Pattern Recognition.

[53] Hirzer M., Beleznai C., Roth P.M., Bischof H. Person re-identification by descriptive and discriminative classification. Proceedings of the Scandinavian Conference on Image Analysis.

[54] Yin J., Li B., Wan F., Zhu Y. A new data selection strategy for one-shot video-based person re-identification. Proceedings of the International Conference on Image Processing.

